# Case report of acute liver injury caused by the eszopiclone in a patient with chronic liver disease

**DOI:** 10.1097/MD.0000000000026243

**Published:** 2021-06-25

**Authors:** Tong Wu, Ge Yu, Zhaoxia Li, Guijie Xin

**Affiliations:** Department of Hepatology, The First Hospital of Jilin University, Changchun, Jilin Province, China.

**Keywords:** case report, chronic liver disease, CYP450, drug-induced liver injury, eszopiclone, trazodone

## Abstract

**Rationale:**

Eszopiclone, sold under the brand name Lunesta, is a new type of non-benzodiazepine hypnotic. Eszopiclone is a zopiclone dextrorotation, which is classified as a cyclopyrrolone. It functions by binding gamma-aminobutyric acid (GABA) receptors. Compared with benzodiazepines hypnotics, eszopiclone has higher selectivity for certain subunits of the GABA(A) receptor. So far, there are no reports about the elevation of serum enzymes or severe liver injury caused by eszopiclone. Here, we present a case report of acute liver injury following eszopiclone treatment in a patient with chronic hepatitis B virus (HBV).

**Patient concerns:**

The patient was a 53-year-old female with a 36-year history of positive HBV markers. Due to poor sleep, the patient took trazodone hydrochloride orally for 1 year. After hospital admission for positive hepatitis B pathogenic markers, abdominal distension, fatigue, and aggravation, she was treated with eszopiclone under the guidance of the mental health department.

**Diagnoses:**

Her transaminase levels increased abnormally after eszopiclone treatment and rapidly decreased after drug withdrawal. This was determined to be an acute liver injury event. liver-protecting treatment was maintained. Considering the patient's anxiety and depression, the patient's family members refused a liver biopsy.

**Outcomes:**

Transaminase levels decreased rapidly within one week, and the patient continued to take trazodone hydrochloride after discharge. No adverse events occurred in the follow-up period.

**Lessons:**

Sleep disorders are more common in patients with chronic diseases, especially patients with chronic liver disease. Recently, it has become common for patients with hepatitis B and C to use antidepressants along with antiviral treatment. Patients with chronic hepatitis B or C may have a threefold risk of liver dysfunction after receiving antituberculosis treatment.^[1,2]^ A proinflammatory environment induced by actively replicating the hepatitis virus may alter the detoxication process and increase drug toxicity.^[3]^ At this time, the safety of other drugs should be reevaluated. Although hepatitis and liver injury are listed as rare adverse reactions of eszopiclone, this case is the first to report the eszopiclone-involved acute liver injury.

## 1 Introduction

Eszopiclone is a fast-acting sedative and hypnotic drug commonly used to prolong sleep time, improve sleep quality, and reduce the number of awakenings after the onset of sleep. Studies have shown that the effective doses of eszopiclone for elderly patients and non-elderly patients to achieve efficient sleep induction and maintenance are 2 and 3 mg, respectively.^[4]^ A systematic review and meta-analysis of a randomized, double-blind, and placebo-controlled trial of eszopiclone in the treatment of primary insomnia showed that eszopiclone is a safe choice for the treatment of primary insomnia, including in elderly patients, and it was also ranked the optimal therapy for prolonging objective total sleep time. Thus far, no reports have been published regarding the elevation of serum enzymes or severe liver injury caused by eszopiclone. The patient in this case report uses eszopiclone in a special disease state. Eszopiclone may aggravate the hepatotoxicity of trazodone through the following mechanisms, which prompted us to pay attention to a series of special populations who also have this risk.

Drug-induced liver injury (DILI) refers to liver disease caused by drugs or metabolites. In Western patients with DILI, antibacterial antibiotics, and psychoactive drugs are the most frequently implicated therapeutic drug classes.^[5]^ Studies have shown that specific patient populations may have an increased risk of DILI. For example, women who use diclofenac^[6]^ and older patients who use isoniazid^[7]^ and amoxicillin-clavulanic^[8]^ both have an increased risk of developing liver damage. Additionally, certain disease states have also been shown to be among the risk factors that can contribute to DILI. These include diabetes and obesity, which are risk factors for methotrexate-induced liver damage, and pre-existing chronic liver disease (CLD), which increases the risk of severe acute liver injury.^[9]^ Therefore, we believe that the special disease state of CLD may also be related to the increased risk of liver damage from certain drugs, although this remains controversial.

In this article, we report a case of acute DILI, determined by acute and transient transaminase elevation, in a 53-year-old woman who was taking drugs for chronic moderate viral hepatitis B, anxiety, depression, and sleep disorders. The cytochrome P450 (CYP) iso-zymes CYP3A4 and CYP2E1 are involved in the bio-transformation of eszopiclone; therefore, drugs that induce or inhibit these CYP isozymes may affect the metabolism of eszopiclone.

## 2 Case report

A 53-year-old woman was admitted to our hospital because of positive hepatitis B pathogenic markers (36 years), abdominal distension, fatigue (1 year), and aggravation (7 days). The patient had a family history of hepatitis B and her mother was an hepatitis B virus (HBV) carrier. For 36 years prior to hospital admission, the patient had taken no hepatitis B medication. In 2001, the patient was diagnosed with “neurosis” due to “anxiety and poor sleep.” She intermittently treated sleep disorders with zolpidem tartrate and other drugs. In May 2018, due to the aggravation of symptoms, the patient began oral administration 150 mg of trazodone hydrochloride until after admission. The patient was monitored for markers related to liver function, including aspartate transaminase (AST), alanine transaminase (ALT), alkaline phosphatase (ALP), gamma-glutamyl transferase, and albumin (ALB). Hepatitis B antigens and HBV-DNA levels were also measured. On November 12, liver function tests showed AST 211.2 U/L, ALT 171.4 U/L, ALP 41.9 U/L, γ-GT 44.7 U/L, and ALB 38.5 g/L (Table 1); HBsAg (+), HBeAg (+), HBeAb (+), and HBcAb (+); HBV-DNA 4.67 × 10^6^ IU/mL; liver transient elastography 8.0 kpa, fat attenuation value 211 db-m; and abdominal computed tomography showed mild fatty liver. Autoimmune antibodies were negative and immunoglobulin levels were normal. The clinical diagnosis was “chronic moderate viral hepatitis B; neurosis.” Each day, the patient was given 15 mL polyene phosphatidylcholine intravenously, 0.5 mg entecavir orally, and continued 150 mg trazodone hydrochloride orally. Re-examination of liver function on November 16 showed AST 197.6 U/L, ALT 91.9 U/L, ALP 39.5 U/L, γ-GT 51.2 U/L, and ALB 31.8 g/L (Table 1).

**Table 1:** Laboratory test results upon patient readmission.

	Date			
Test parameter	12 November	16 November	24 November	2 December
AST^1^(U/L)(15–40)	211.2	197.6	2969	194.2
ALT^2^(U/L)(9–50)	171.4	91.9	203.7	80.1
γ-GT^3^(U/L)(10–60)	44.7	51.2	204.7	415.3
ALP^4^(U/L)(45–125)	41.9	39.5	54	88
ALB^5^(g/L)(40–55)	38.5	31.8	32.2	26.9
HBV-DNA(IU/mL)(<50)	4.67 × 10^6^			2.76 × 10^4^

Provides the laboratory test results of liver function after admission. The test date is selected based on the improvement of the patient's symptoms, such as fatigue and loss of appetite. γ-GT = γ-glutamyl transpeptidase, ALB = albumin, ALP = alkaline phosphatase, ALT = alanine aminotransferase, AST = aspartate aminotransferase.aspartate aminotransferase.alanine aminotransferase.γ-glutamyl transpeptidase.alkaline phosphatase.albumin.

On November 22, the patient's sleep disorder worsened, and the Department of Mental Health recommended adding 3 mg eszopiclone orally each day. Re-examination of liver function on November 24 showed AST 2969 U/L, ALT 203.7 U/L, ALP 54U/L, γ-GT 204.7 U/L, and ALB 32.2 g/L (Table 1). We immediately stopped administering eszopiclone and applied hepatoprotective drugs, which maintain the previous treatment plan, that is,15 mL of polyene phosphatidylcholine is given daily by intravenous infusion. Another liver function test on December 2 showed AST 194.2 U/L, ALT 80.1 U/ L, ALP 88 U/L, γ-GT 415.3 U/L, ALB 26.9 g/L, and HBV-DNA 2.76 × 10^4^ IU/mL (Table 1). The liver enzyme index decreased significantly by December 2. In the process of the increase and decrease of transaminase, the patient has no obvious symptom changes. Table 1 provides the laboratory test results of liver function after admission. The test date is selected based on the improvement of the patient's symptoms, such as fatigue and loss of appetite.

## 3 Discussion

Eszopiclone, a new type of non-benzodiazepine hypnotic, is a zopiclone dextrorotation, which is classified as a cyclopyrrolone. Its affinity for non-benzodiazepine receptors is much stronger than that of zopiclone levorotatory. Additionally, it is more efficient at inducing sleep, reducing arousal, and reducing anxiety compared with zopiclone. It is a gamma-aminobutyric acid/BZI receptor complex, which can achieve sedative and hypnotic effects by inhibiting the excitatory center. The peak blood concentration occurs at about 1 hour, and the half-life is only about 6 hours. Compared with other types of sedative-hypnotics, it has a more rapid onset and fewer side effects. It improves sleep quality without damaging psychomotor function, generally without drug dependence, and it can significantly shorten the patient's sleep latency, prolong the patient's sleep time, and improve sleep quality.^[10]^ Although hepatitis and liver injury are listed as rare adverse reactions on the product label, since the approval and widespread use of eszopiclone, there has been no relevant report on clinically obvious liver disease caused by eszopiclone.

This patient in this case report had preexisting CLD (viral hepatitis B). Before admission, she took trazodone regularly for 1 year. During hospitalization, she was given antiviral therapy; 10 days later, eszopiclone was added. Following treatment with eszopiclone, transaminase increased significantly and then decreased rapidly after drug withdrawal. Based on ALT ≥3 times the upper limit of normal (ULN) and *R* value [(ALT/ULN)/(ALP/ULN)] ≥5, the patient was diagnosed with the hepatocellular injury.^[11,12]^ Furthermore, the Roussel Uclaf Causality Assessment Method (RUCAM) was used to evaluate the case. The RUCAM score is was 6 points after applying eszopiclone, meaning that eszopiclone was categorized as the probable cause of liver injury.^[13]^ Due to the poor condition of the patients themselves and the high transaminase levels during hospitalization, the re-drug reaction test could not be performed. The score was affected by various factors without further examination and verification.

The difficulty of this case is that the patient had basic liver disease, and acute liver function injury often occurs in the active stage of chronic hepatitis. The incidence of CLD in the general population is on the rise, presenting challenges for clinicians diagnosing DILI in these patients. In this case report, the liver injury was very likely related, either directly or indirectly, to the patient's use of eszopiclone. However, due to the patient's anxiety, it was impossible to obtain a liver biopsy for pathological auxiliary diagnosis.

This also reminds us that patients with liver disease are in a special immune state, and the related medication safety requires our close attention. A systematic review and meta-analysis of symptoms, morbidity, and quality of life of end-stage liver disease show that the incidence of depression, anxiety, and sleep disorders in these patients is 4.5% to 64%, 14% to 45%, and 26% to 77%, respectively.^[14]^ The incidence of these disorders correlates with the economic burden of liver disease patients and the deterioration of social and occupational function. Some studies have shown that metabolic disorders of melatonin and glucose, changes in thermoregulation, and secretion characteristics of ghrelin may be related to sleep disorders in patients with CLD,^[15]^ and compared with chronic hepatitis B patients, patients with cirrhosis and acute-on-chronic liver failure have a higher proportion of insomnia, which may be attributed to the more serious physical symptoms of these diseases.^[16,17]^ At present, new drugs for the treatment of anxiety, depression, and sleep disorders emerge continually, which makes it challenging to ascertain the safety and efficacy of these drugs used by patients with liver disease.

The patient, in this case, used trazodone long-term use for anxiety and depression. Trazodone is a triazole pyridine derivative, which is a new type of antidepressant used to treat depression, aggressive behavior, and panic disorder. Trazodone can be associated with transient, usually asymptomatic, elevated serum transaminase levels. The injury usually occurs after several months of continuous ingestion and it usually manifests as elevated transaminase. It is also associated with rare cases of acute liver injury in clinical practice.^[18,19]^ Studies have shown that CYP3A4 and CYP2D6 inhibitors can affect the cytotoxicity of trazodone, suggesting that trazodone-induced liver cytotoxicity is at least partially induced by its metabolites.^[20,21]^ The cytochrome P450 CYP3A4 is exclusively involved in the metabolism of trazodone in the human body.^[22]^ This enzyme plays a key role in the metabolic activation of trazodone,^[21]^ and eszopiclone is also metabolized by cytochrome P450s (mainly CYP3A4 and CYP2E1).^[23]^ The effect of eszopiclone or its metabolites on CYP3A4 may be an important reason for the aggravation of trazodone hepatotoxicity by eszopiclone. It is worth noting in this case that the patient had chronic HBV infection and had not received standard treatment for many years. After hospitalization, the patient has prescribed the antiviral entecavir. Some studies have shown that HBV infection and the use of antiviral drugs can inhibit the activities of CYP2C9 and CYP3A4, cause abnormal drug metabolism, and change the blood drug concentration, which can also cause acute liver injury.^[24]^

The treatment of various types of liver diseases has recently made great progress, but there are still many patients with end-stage liver disease who need our attention, especially regarding the improvement of quality of life and drug safety. At present, there is no comprehensive study of antidepressant and anxiety drugs in the treatment of sleep disorders in such patients. It is critical to understand these drugs in the context of liver disease to best care for these patients.

## Author contributions

**Funding acquisition:** Ge Yu, Guijie Xin.

**Investigation:** Zhaoxia Li.

**Resources:** Guijie Xin.

**Supervision:** Ge Yu, Guijie Xin.

**Validation:** Guijie Xin.

**Writing – original draft:** Tong Wu.

**Writing – review & editing:** Tong Wu.
